# Cardiac taurine and principal amino acids in right and left ventricles of patients with either aortic valve stenosis or coronary artery disease: the importance of diabetes and gender

**DOI:** 10.1186/2193-1801-3-523

**Published:** 2014-09-13

**Authors:** Martin Lewis, Ben Littlejohns, Hua Lin, Gianni D Angelini, M-Saadeh Suleiman

**Affiliations:** Bristol Heart Institute, University of Bristol, Bristol, UK; Bristol Heart Institute, Bristol Royal Infirmary, Level 7, Bristol, BS2 8HW UK

**Keywords:** Left and right ventricle, Aortic valve stenosis, Coronary artery disease, Hypertrophy, Taurine, Glutamine, Glutamate, Alanine

## Abstract

Free intracellular taurine and principal α-amino acids (glutamate, glutamine, aspartate, asparagine and alanine) are abundant in human heart. They are cellular regulators and their concentration can change in response to disease and cardiac insults and have been shown to differ between hypertrophic left ventricle (LV) and the relatively “normal” right ventricle (RV) in patients with aortic valve stenosis (AVS). This difference has not been shown for coronary artery disease (CAD) and there are no studies that have simultaneously compared amino acid content in LV and RV from different pathologies. In this study we investigated the effect of disease on taurine and principal amino acids in both LV and RV, measured in myocardial biopsies collected from patients with either AVS (n = 22) or CAD (n = 36). Amino acids were extracted and measured using HPLC. Intra- and inter-group analysis was performed as well as subgroup analysis focusing on gender in AVS and type 2 diabetes in CAD. LV of both groups has significantly higher levels of taurine compared to RV. This difference disappears in both diabetic CAD patients and in male AVS patients. Alanine was the only α-amino acid to be altered by diabetes. LV of female AVS patients had significantly more glutamate, aspartate and asparagine than corresponding RV, whilst no difference was seen between LV and RV in males. LV of females has higher glutamate and glutamine and less metabolic stress than LV of males. This work shows that in contrast to LV, RV responds differently to disease which can be modulated by gender and diabetes.

## Introduction

Cardioprotective strategies against ischemia and reperfusion are aimed at preserving intracellular metabolites and opposing disruption to ionic homeostasis. Evaluation of the efficacy of cardioplegic solutions during open heart surgery has relied upon monitoring intracellular changes of key metabolites including amino acids (Caputo et al. 1998a, [b]; Suleiman et al. 2001, 2008; Venturini et al. 2009). During cardioplegic arrest, the heart is rendered ischemic and therefore vulnerable to reperfusion injury caused by Ca^2+^ loading and generation of reactive oxygen species (Suleiman et al. 2008). Ca^2+^ loading occurs as a consequence of ischemia-induced Na^+^ accumulation. In addition, Na^+^ accumulation contributes to osmotic-induced cell swelling, which has been implicated as a cause of ischemia-induced sarcolemmal damage (Garcia-Dorado and Oliveras 1993; Ruiz-Meana et al. 1995; Tritto et al. 1998). In response to cardiac insults/osmotic stress, heart cells release free amino acids including the non-protein β-amino acid taurine (Schaffer et al. 2002; Suleiman and Chapman 1993a, [b]; Suleiman et al. 1993). Taurine is the main organic osmolyte in the heart (Huxtable 1993) and its trans-sarcolemmal fluxes are dependent on the Na^+^ gradient (Suleiman et al. 1992). Our work and those of others has shown that the fall in tissue taurine is due to efflux as it is detected in the effluent of hearts artificially perfused *ex vivo* (Suleiman et al. 1992). This is consistent with taurine being very slowly metabolized and the finding that its concentration is raised in the blood of patients following acute myocardial infarction, unstable angina, and cardiovascular surgery (Cooper and Lombardini 1981; Lombardini and Bricker 1981; Lombardini and Cooper 1981). This close link between ischemic cardioplegic stress and taurine has been supported by our work involving patients undergoing open heart surgery (Ascione et al. 1998; Caputo et al. 1998a, [b]; Lotto et al. 2003; Suleiman et al. 1993, 1997) where we have demonstrated that the higher the metabolic stress, the greater the fall of myocardial taurine during reperfusion. Extensive studies have been carried out establishing a link between taurine and type 2 diabetes largely in the context of using taurine as an additive, monitoring taurine plasma levels and depleting or replacing taurine with an array of compounds (e.g. Das et al. 2012; Gossai and Lau-Cam 2009; Tappia et al. 2011, 2013). The majority of studies suggest beneficial effects of exogenous taurine in protecting the diabetic cardiomyopathic heart as diabetic patients have lower plasma taurine levels (Franconi et al. 1995, 2004). Whether lower plasma taurine reflects an increased uptake by diabetic ventricle(s) is not presently known.

In addition to taurine, the principal free α-amino acids glutamine, glutamate, aspartate and alanine also change in heart cells during cardiac insults in both experimental models and during open heart surgery (Suleiman and Chapman 1993b; Suleiman et al. 1993, 1997). However, unlike taurine the changes in these amino acids involve metabolism and transport and influence the biosynthesis of several key compounds including energy rich phosphates (Rennie et al. 2001). The extent of the changes in cardiac amino acids during ischemia and reperfusion depends on their basal level which is determined both by disease state and whether the tissue comes from LV or RV. For example in hearts with aortic valve stenosis, pressure overload triggers structural, functional and metabolic remodeling in the LV (Petrov et al. 2010). In contrast and in the early stages of the disease, the RV is not affected by these compensatory mechanisms, as it is not chronically overloaded. As a result, the cardiac pump is likely to end up having two ventricles with different metabolic states. Left ventricular inter-disease (coronary vs. valve) differences in the content of amino acids have also been reported (Suleiman et al. 1998).

Interestingly, there are no studies that simultaneously compare cardiac amino acids in LV and RV of both coronary artery disease and aortic valve stenosis patients. We have shown that the two pathologies required different cardioplegic interventions to achieve optimal cardio-protection (Suleiman et al. 2001, 2008, 2011). We now propose that the design of cardio-protective strategies should also take into account the fact that the two sides of the heart are different and may respond differently to ischemia and reperfusion. To address this point we measured myocardial amino acids in both LV and RV of patients with either aortic valve stenosis (AVS) or coronary artery disease (CAD).

## Materials and methods

### Patients

Adult patients (n = 58) having either elective or urgent isolated coronary artery bypass grafting or aortic valve replacement using cardiopulmonary bypass were included in this study. Exclusions included patients who had undergone previous surgery, were having combined coronary and valve surgery, an emergency or salvage operation, were already participating in another clinical trial, or those with chronic renal failure requiring dialysis, current congestive heart failure or poor left ventricular function.

The investigation conforms to the principles outlined in the Declaration of Helsinki. Ethical approval by the NHS Research Ethics Committee (ISRCTN84968882) as well as patient consent were obtained. Patients with coronary artery disease (included only 3 female patients) were analyzed for the effect of type 2 diabetes whilst patients with aortic valve disease (included only one diabetic patient) were analyzed for the effect of gender. Patients’ characteristics are shown in Table 1. There were no significant differences in terms of NYHA classification between the subgroups. The number of female patients with CAD undergoing surgery (9%) is relatively small compared to approximately 32% reported by others (Blankstein et al. 2005). However, CAD females very often present with other co-morbidities which is in contrast to this study where surgery was only for isolated CAD. The incidence of diabetes mellitus in patients undergoing aortic valve surgery tends to be low and similar for both males and females at approximately 10% (Higgins et al. 2011).

**Table 1:** **Patients characteristics**

	Coronary artery disease		
	Total (n = 36)	Diabetics (n = 13)	Non-diabetic (n = 23)
Age (years)	67.1 ± 1.3	66.2 ± 2.4	67.6 ± 1.6
Sex (male/female)	33/3	13/0	20/3
NYHA Classification			
I	10	5	5
II	16	5	11
III	9	3	6
IV	1	0	1
	**Aortic Valve Disease**		
	**Total (n = 22)**	**Male (n = 10)**	**Female (n = 12)**
Age (years)	68.9 ± 2.0	65.0 ± 3.3	72.1 ± 2.0
Diabetes (Yes/No)	1/21	0/10	1/11
NYHA Classification			
I	4	2	2
II	7	3	4
III	11	5	6
IV	0	0	0

Data are shown as mean ± SEM. NYHA = New York Heart Association.

### Collection of ventricular biopsies

Patients were anesthetized in the routine manner used in our institute, and then cardiopulmonary bypass was initiated. Immediately following the institution of cardiopulmonary bypass, myocardial tissue biopsy specimens were collected from the apex of LV and RV using a 14 Ga. TW’11.4 cm cannula Trucut needle (Baxter Healthcare Corporation, USA). Each specimen was immediately snap frozen (less than 5 s) in liquid nitrogen and stored at -80°C until processing for amino acid extraction and quantification.

### Determination of amino acids in biopsy specimens

Free amino acids were measured in all biopsies collected. Amino acids were determined using HPLC according to the Pico-Tag method of Water as reported earlier (Caputo et al. 1998b). In brief, frozen biopsy specimens were crushed in liquid nitrogen and the resultant powder was extracted with 4.8% perchloric acid. Neutralized 100 μL of the extract was dried immediately after extraction using vacuum centrifugation (Savant SV160). Free amino acids were derivatized by phenylisothiocyanate (PITC) and were separated by 30 cm Pico-Tag column (Millipore Corporation, USA) with two Beckman delivery pumps at a constant flow rate of 1 ml/min with gradient elution for 25 min at 46°C. Derivatized amino acids were detected at 254 nm using a Beckman System Gold 166 Detector. Quantitative analysis was carried out using amino acid standards (Thermo, UK) and the acquired data were processed using 32 Karat software supplied by Beckman. Concentration of metabolites was expressed as nmol/mg wet weight.

### Statistical analysis

Data were expressed as mean ± standard error of the mean (SEM). Comparison between continuous variables was made using non-parametric tests for paired or unpaired samples (tested using Mann–Whitney or Wilcoxon’s signed rank). All statistical analyses mentioned were performed with the aid of a computerized software package, Statview for Windows (SAS Institute Inc., NC, USA).

## Results

### The effect of CAD compared to AVS on levels of amino acids in LV and RV

**Figure 1:**
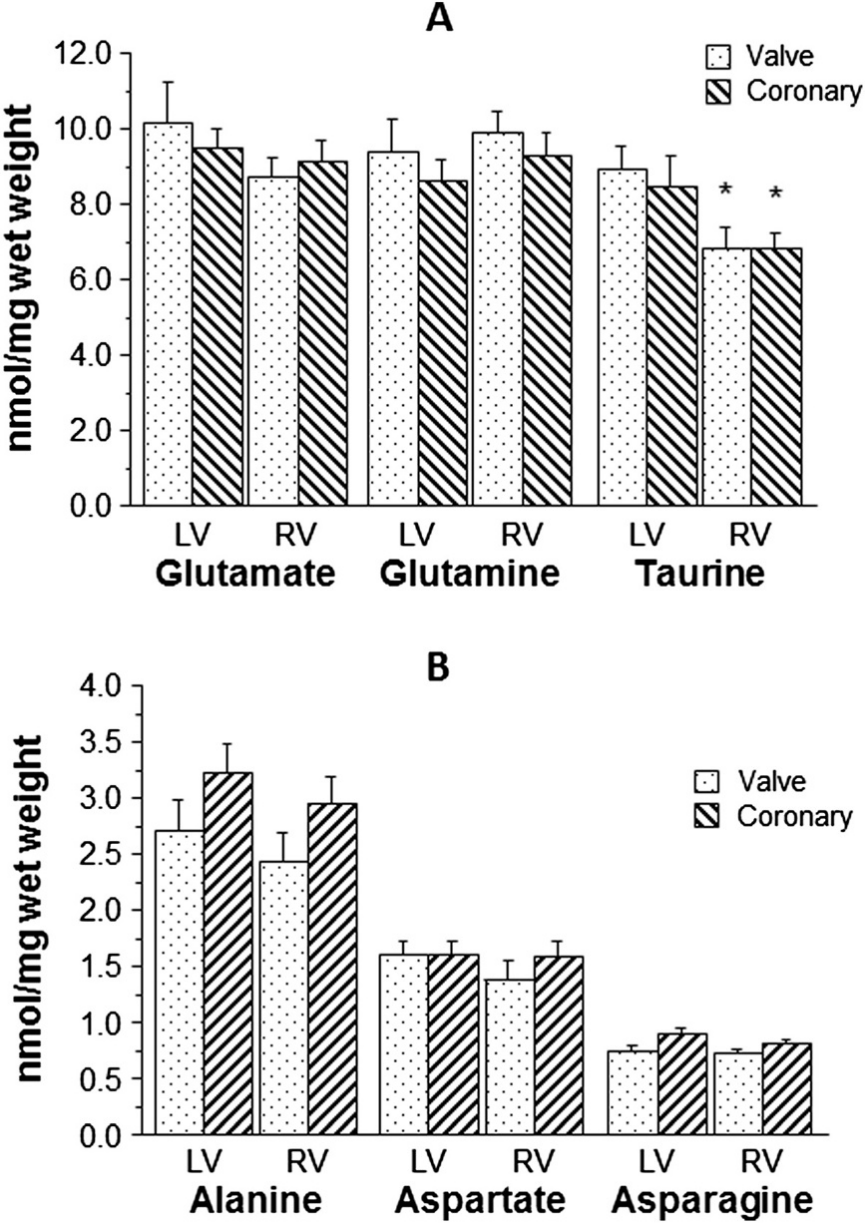
**Effect of anatomy and pathology on basal levels of cardiac amino acids.** The myocardial concentration of taurine and free principal α-amino acids (glutamate, glutamine, alanine, aspartate and asparagine) in biopsies collected from both left (LV) and right (RV) ventricles of hearts of aortic valve stenosis (n = 22) and coronary artery disease (n = 36) patients. Panel **(A)** shows data for taurine, glutamine and glutamate while panel **(B)** shows data for alanine, aspartate and asparagine. Data are shown as mean ± SEM. * = p < 0.05 vs. corresponding left ventricle.

There was no difference in the cardiac α-amino acids, glutamate, glutamine, aspartate, asparagine and alanine between LV and RV of CAD patients (Figure 1A-B). However, the β-amino acid taurine was significantly lower in RV compared to the LV (6.8 ± 0.4 vs. 8.5 ± 0.8 nmol/mg wet weight, p < 0.05). There was no difference in the protein α-amino acids between the LV and RV of AVS patients whilst taurine was significantly lower in the RV compared to the LV (6.8 ± 0.5 vs. 8.9 ± 0.6 nmol/mg wet weight, p < 0.05). Comparison between CAD and AVS for LV and RV did not show any significant difference. However, alanine tended to be higher in LV and RV of CAD hearts compared to AVS (LV: 3.2 ± 0.2 vs. 2.7 ± 0.3 nmol/mg wet weight and RV: 3.0 ± 0.2 vs. 2.4 ± 0.2 nmol/mg wet weight) (Figure 1B).

### Effect of diabetes on levels of amino acids in LV and RV of patients with CAD

**Figure 2:**
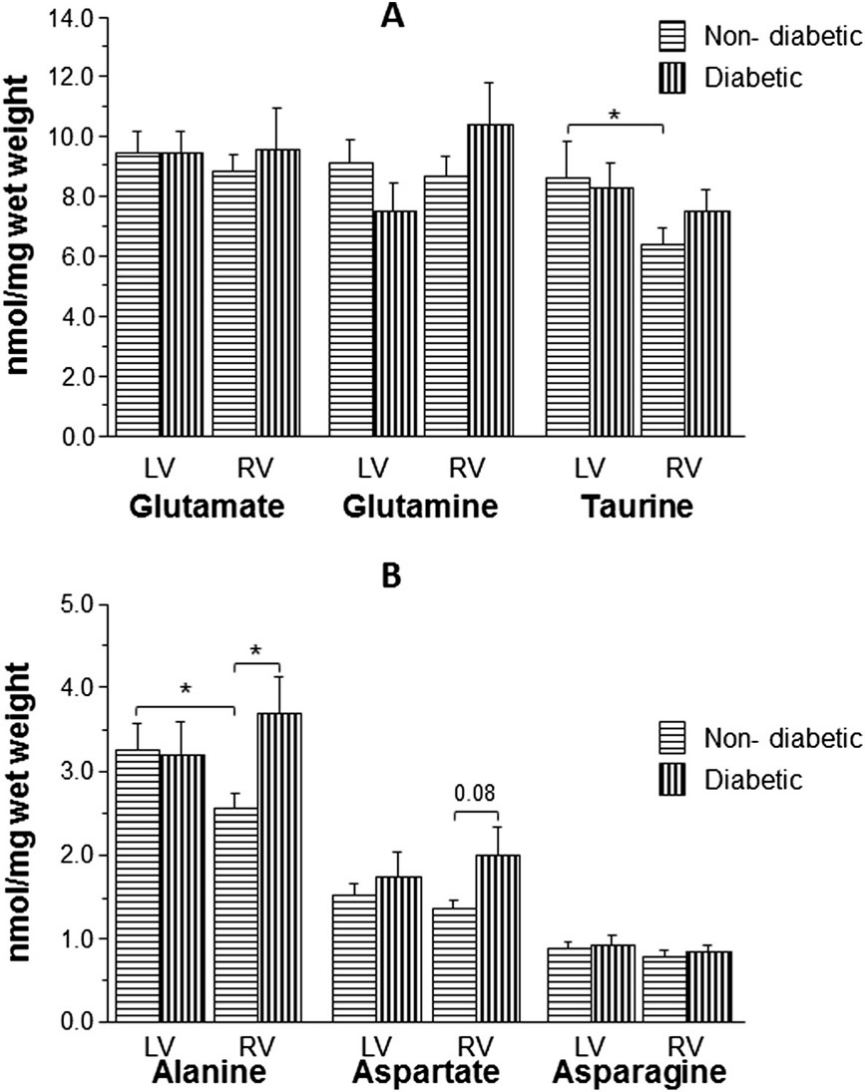
**Effect of diabetes on basal levels of amino acids in left and right ventricles of coronary artery disease patients.** The concentration of taurine and principal free amino acids (glutamate, glutamine, alanine, aspartate and asparagine) in biopsies collected from both left (LV) and right (RV) ventricles of diabetic (n = 13) and non-diabetic (n = 23) coronary artery disease patients. Panel **(A)** shows data for taurine, glutamine and glutamate while panel **(B)** shows data for alanine, aspartate and asparagine. Data are shown as mean ± SEM. * = p <0.05.

Both taurine and alanine were significantly lower in RV compared to LV in non-diabetic patients (Taurine: 6.4 ± 0.5 vs. 8.6 ± 1.2 nmol/mg wet weight and alanine: 2.5 ± 0.2 vs. 3.2 ± 0.3 nmol/mg wet weight, p < 0.05) (Figure 2A-B). In contrast, diabetic patients had similar levels of taurine and alanine in both LV and RV. The RV of diabetic patients had significantly higher levels of alanine compared to RV of non-diabetic patients (3.7 ± 0.5 vs. 2.5 ± 0.2 nmol/mg wet weight, p < 0.05) (Figure 2B). A similar trend was seen for aspartate and glutamine.

### Effect of gender on levels of amino acids in LV and RV of patients with AVS

**Figure 3:**
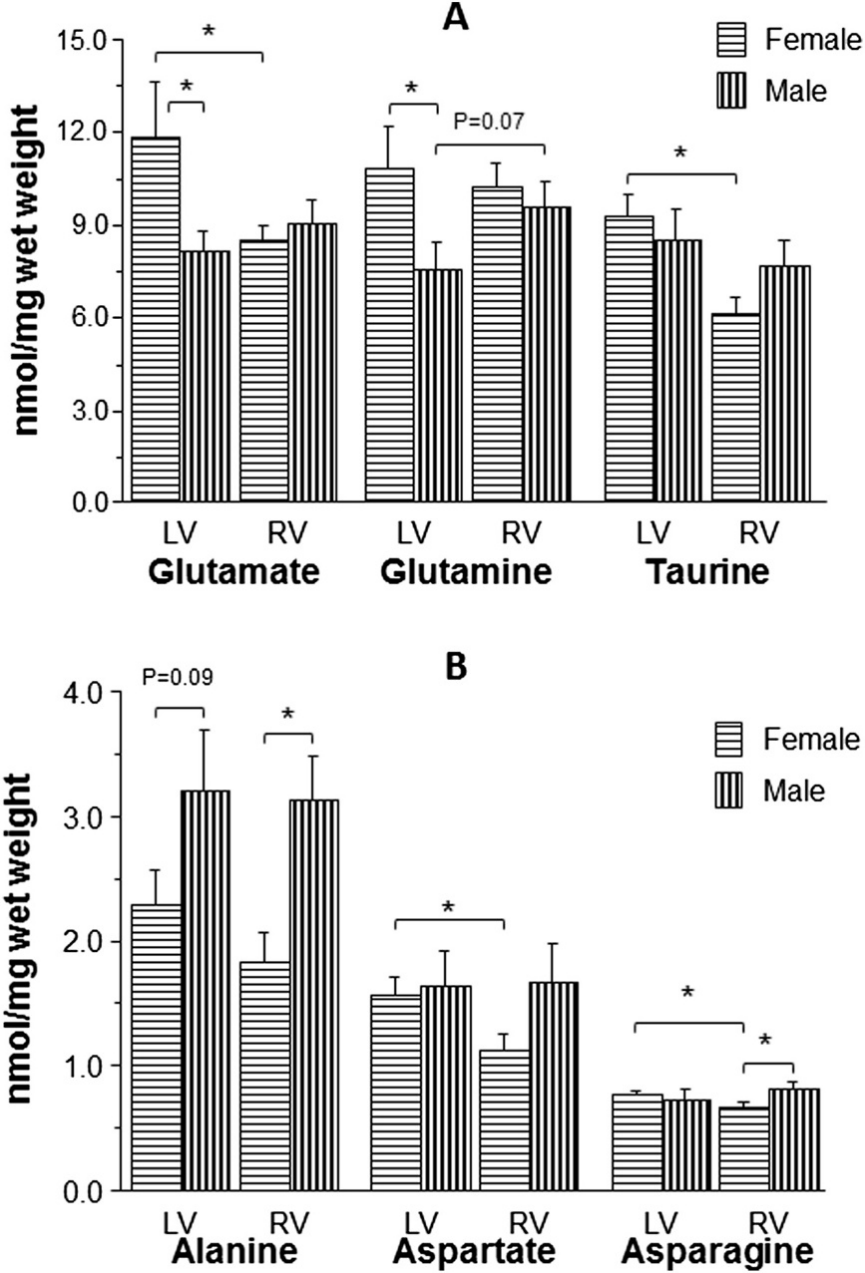
**Effect of gender on basal levels of amino acids in left and RVs of aortic valve stenosis patients.** The concentration of taurine and principal free amino acids (glutamate, glutamine, alanine, aspartate and asparagine) in biopsies collected from both left (LV) and right (RV) ventricles of male (n = 10) and female (n = 12) aortic valve stenosis patients. Panel **(A)** shows data for taurine, glutamine and glutamate while panel **(B)** shows data for alanine, aspartate and asparagine. Data are shown as mean ± SEM. * = p <0.05.

Apart from a strong trend for glutamine to be lower in the LV compared to the RV, there was no difference in amino acids, including taurine, between LV and RV for male patients (Figure 3A-B). In contrast taurine, glutamate, aspartate and asparagine were significantly higher in the LV compared to RV of female patients. Alanine was higher in both LV and RV of males compared to females (LV: 3.2 ± 0.5 vs. 2.3 ± 0.3 nmol/mg wet weight and RV: 3.1 ± 0.4 vs. 1.8 ± 0.2 nmol/mg wet weight) (Figure 3B).

### Effect of disease, gender and diabetes on metabolic activity/stress in LV and RV

**Figure 4:**
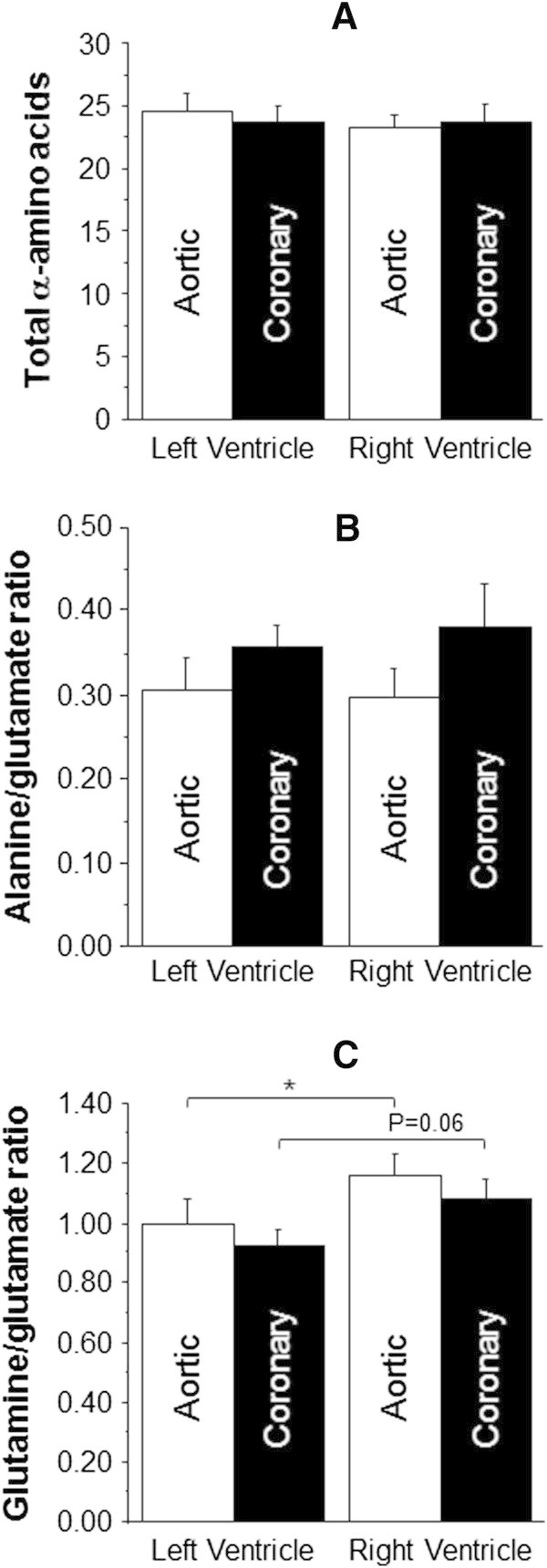
**The effect of disease on markers of stress in left and RVs.** Total free α-amino acids **(A)**, alanine/glutamate ratio **(B)** and glutamine/glutamate ratio **(C)** in biopsies collected from both left and RVs of hearts of aortic valve stenosis (n = 22) and coronary artery disease (n = 36) patients. Data are shown as mean ± SEM. * = p <0.05.

**Figure 5:**
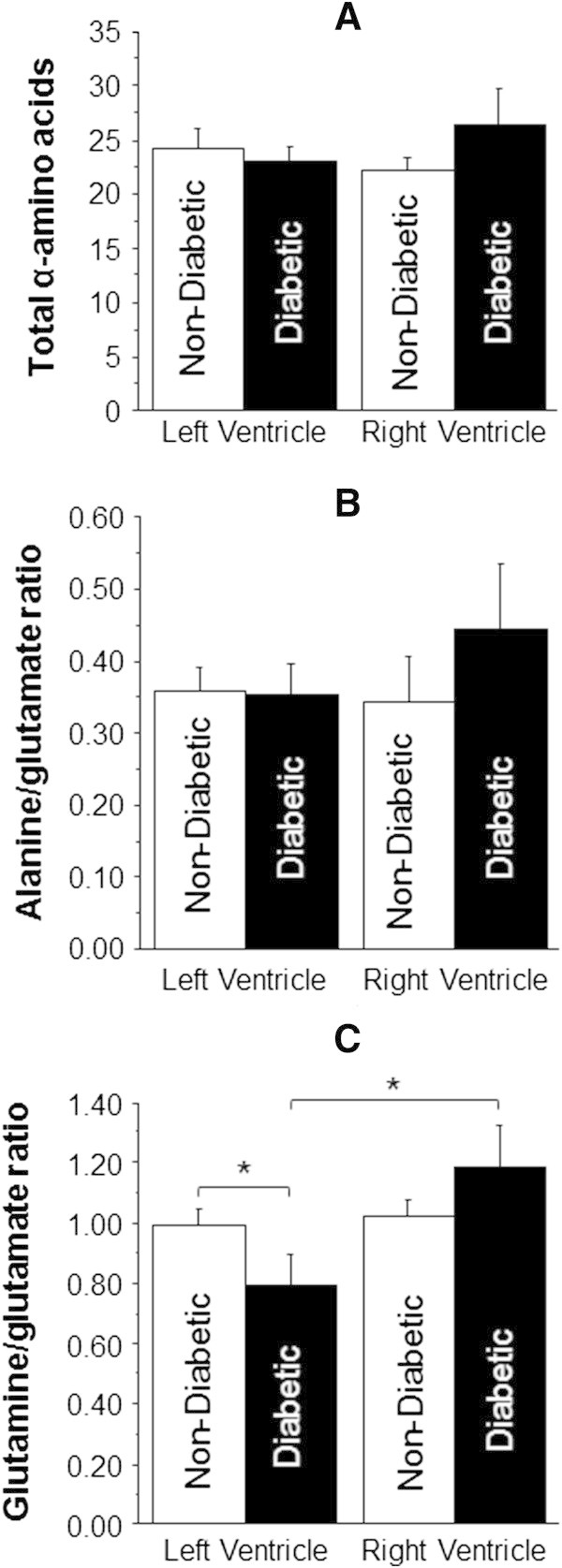
**The effect of diabetes in coronary artery disease on markers of stress in left and RVs.** Total free α-amino acids **(A)**, alanine/glutamate ratio **(B)** and glutamine/glutamate ratio **(C)** in biopsies collected from both left and RVs of diabetic (n = 13) and non-diabetic (n = 23) coronary artery disease patients. Data are shown as mean ± SEM. * = p <0.05.

**Figure 6:**
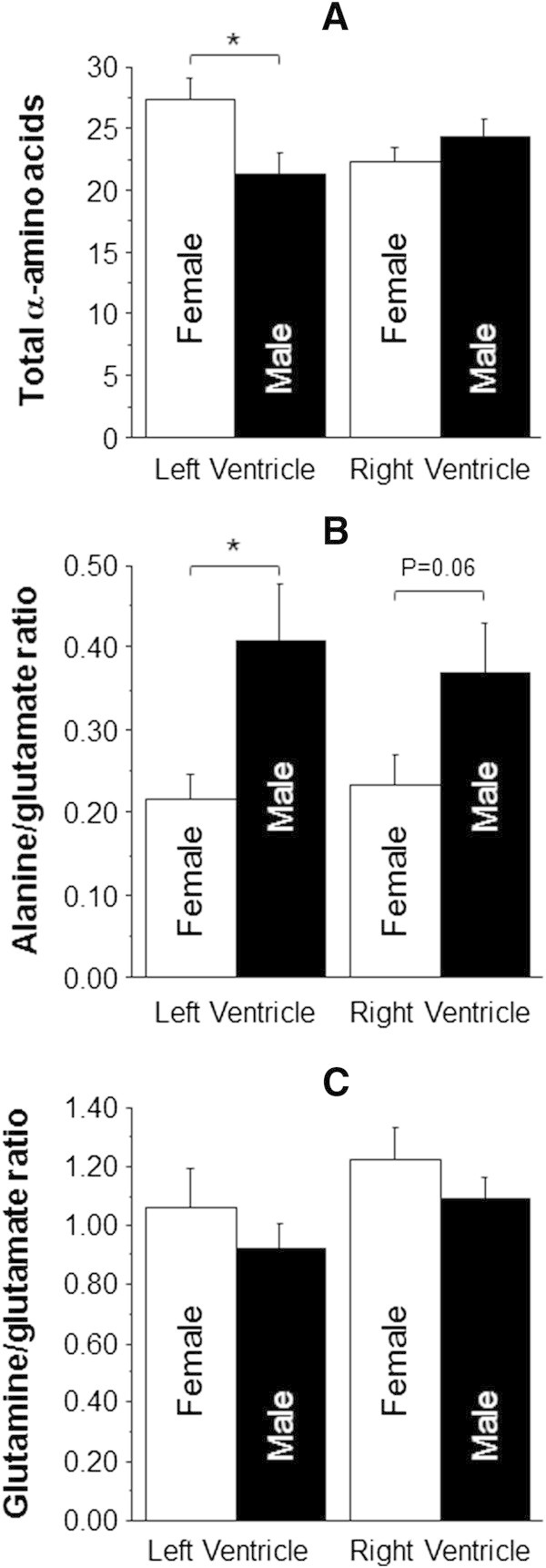
**The effect of gender in aortic valve stenosis on markers of stress in left and RVs.** Total free α-amino acids **(A)**, alanine/glutamate ratio **(B)** and glutamine/glutamate ratio **(C)** in biopsies collected from both left and RVs of male (n = 10) and female (n = 12) aortic valve stenosis patients. Data are shown as mean ± SEM. * = p <0.05.

Figure 4 shows the effect of disease on total α-amino acids and markers of metabolic stress (ratios of alanine/glutamate and glutamine/glutamate). Both LV and RV of CAD tended to show higher evidence of metabolic stress (alanine/glutamate) compared to aortic valve disease (LV: 0.36 ± 0.03 vs. 0.30 ± 0.04 nmol/mg wet weight and RV: 0.38 ± 0.05 vs. 0.30 ± 0.04 nmol/mg wet weight). Furthermore, the glutamine/glutamate ratio was significantly higher in the RV compared to LV of each pathology (Figure 4C).Figure 5 shows data from CAD comparing the effect of diabetes. The diabetic RV tended to have bothelevated total α-amino acids and a higher alanine/glutamate ratio compared to both diabetic LV and non-diabetic RV. The diabetic LV had significantly lower glutamine/glutamate ratio compared to diabetic RV (0.80 ± 0.10 vs. 1.19 ± 0.14 nmol/mg wet weight, p < 0.05). The diabetic LV had a significantly lower glutamine/glutamate ratio compared to non-diabetic LV (0.80 ± 0.10 vs. 1.00 ± 0.06 nmol/mg wet weight, p < 0.05).Figure 6 shows the data from male and female patients with AVS. The total α-amino acids (Figure 6A) were higher in the LV of female patients compared to other ventricles, although this was only significant compared to male LV (27.3 ± 1.7 vs. 21.3 ± 1.7 nmol/mg wet weight, p < 0.05). The male LV had significantly more metabolic stress/activity compared to female LV (0.41 ± 0.07 vs. 0.22 ± 0.03 nmol/mg wet weight, p < 0.05) and a trend to increase in the RV (0.37 ± 0.06 vs. 0.23 ± 0.04 nmol/mg wet weight).

## Discussion

To the best of our knowledge, this is the first study that simultaneously compares the myocardial concentration of taurine and the principal α-amino acids in LV and RV of patients with either CAD or AVS. Previous studies tended to focus on one pathology and/or changes in one ventricle. In the present study, we show the novel observations that the LV of either coronary artery disease or aortic valve disease patients has significantly higher levels of taurine compared to their respective RV and that this difference disappears in both diabetic patients with coronary artery disease and in male patients with aortic valve disease. Diabetic RV and non-diabetic LV have more alanine compared to non-diabetic RV. This is consistent with stress in diabetic RV as shown by a higher glutamine/glutamate ratio. The two chambers of the heart are different in females but not males. The LV of female patients had significantly more glutamate, aspartate, asparagine than corresponding right and more glutamate and glutamine than LV of male patients. This LVs of female patients have relatively less metabolic stress as measured by a low alanine/glutamate ratio.

### Left ventricle has more taurine than the right ventricle irrespective of disease state and this difference is dependent on diabetes and gender

Taurine concentration, measured simultaneously in both the left and right ventricles of the two cardiac diseases considered is higher in the left ventricle than in the right ventricle. The levels and the extent of the differences were similar for both groups of patients. This suggests that the left and right ventricles have different taurine levels irrespective of the disease state. The finding that taurine is higher in the LV of patients with AVS has already been reported (Lotto et al. 2003). The LV of these patients is hypertrophic and the RV is relatively normal. However, a similar observation has also been made for patients with mitral valve disease where the LV is dilated (Venturini et al. 2009). In this work, we show for the first time that patients with coronary artery disease also have higher taurine levels in their LV compared to RV. These hearts, and unlike hearts from patients with aortic valve disease, do not have a homogeneous disease-induced global remodeling (e.g. left ventricular hypertrophy, in the case of aortic valve disease). In patients with coronary artery disease (significant occlusive atherosclerosis), the macroscopic effect of disease is in most cases largely restricted to a small number of vessels. However, the availability of coronary flow reserve can compensate for the effect of stenosis by way of vasodilation of the downstream arteriolar resistance vessels which will help to maintain coronary flow in these areas (Di Carli et al. 1995; Gould 2005; Gould et al. 1975, 2000). Additionally, the biopsies were collected from areas distal to the stenosis. Therefore, LV with global remodeling (hypertrophy or dilation) appears to be similar to LV that has localized atherosclerotic disease. What is interesting however, is the finding that a dilated LV secondary to mitral valve disease has a markedly higher taurine concentration (1.55 fold) compared to right ventricle (Venturini et al. 2009). In contrast, taurine in the hypertrophic LV secondary to aortic stenosis is only 1.26–1.32 fold higher than right ventricle ((Lotto et al. 2003) and Figure 1A). This is similar or slightly higher than coronary artery disease patients with 1.25 fold (Figure 1A). This is not straight forward as our study revealed for the first time that taurine content in left and right ventricles of patients with coronary artery disease is dependent on diabetes.

Diabetic patients with coronary artery disease have similar levels of taurine in both ventricles whereas non-diabetics have ~1.3 fold more taurine in the LV compared to the right ventricle. This is largely due to a trend for the right ventricle of diabetics to have more taurine than the right ventricle of non-diabetics (Figure 2A). Diabetes and associated cardiovascular complications have been linked to changes in plasma and myocardial taurine levels. Diabetes leads to depletion of intracellular taurine (Hansen 2001) which appears to be associated with the development of late diabetic cardiomyopathy (Li et al. 2005). Additionally diabetic patients have significantly lower plasma taurine than normal subjects (Franconi et al. 1995). It is not, therefore, surprising that dietary supplementation of taurine attenuates diabetes-induced changes in cardiac contractile function and ultrastructure (Das et al. 2012; Tappia et al. 2011). Interestingly, the depletion of taurine has been linked to the development of atherosclerosis (Hansen 2001). Since both diabetics and non-diabetics have coronary artery disease, it is likely that the reported diabetes-induced lower taurine in myocardium is due to diabetic cardiomyopathy independent of coronary artery disease. The lower plasma levels in diabetic patients could be a consequence of tissue accumulation as seen here for the right ventricle. In the context of taurine having major cellular effects during cardiac insults, this work suggests that LV of diabetics is likely to show similar vulnerability to reperfusion injury compared to non-diabetics. However, the right ventricle of non-diabetics with low taurine could be either preconditioned and more resistant to insults (Allo et al. 1997) or more vulnerable due to less available taurine to help efflux accumulated Na^+^ and to oppose oxidative stress and apoptotic death (Chapman et al. 1993; Suleiman et al. 1992) (Parildar et al. 2008; Schaffer et al. 2010, 2014; Takahashi et al. 2003; Takatani et al. 2004a, [b]). Interestingly, taurine deficiency, rather than accumulation, has been implicated in the development of cardiomyopathy (Ito et al. 2008) and therefore it can be argued that taurine accumulation is in fact an adaptation to disease development. This is supported by an earlier finding in dogs with congestive heart failure where the severity of the disease significantly correlated with taurine content (Newman et al. 1977). It must be stressed however, that taurine depletion is also associated with adaptation to pathology as neonatal myocytes depleted of taurine showed significant changes in shape and size (Schaffer et al. 1998). Clearly more work is needed to provide an answer to this issue.

Left to right ventricle taurine ratio in female patients with aortic valve stenosis was ~1.55 which is similar to patients with mitral valve disease (see above). On the other hand taurine levels were similar for both left and right ventricles of male patients (~1.1). The relatively lower level of taurine in the right ventricle of female patients with aortic valve stenosis is similar to the finding for non-diabetic right ventricle of coronary artery disease patients (see above).

### Alanine is the only α-amino acid that changes in response to coronary artery disease and diabetes

There was no intra- or inter-disease differences in any of the principal α-amino acids (Figure 1). Our earlier finding that the LV of patients with coronary artery disease had significantly higher alanine levels than patients with aortic valve stenosis (Suleiman et al. 1998) has not been confirmed in this study. However, there was a strong tendency for alanine to be higher in both left and right ventricles of coronary artery disease hearts compared to aortic valve disease (Figure 1B). A likely explanation for this could be the disease intensity which if not severe could mask the difference as the difference was attributed to higher ischemic stress due to coronary artery disease. However, a closer look shows that the trend to accumulate alanine in the right ventricle appears to be associated with diabetes (Figure 2B). This is consistent with stress in diabetic right ventricle as shown by a higher glutamine/glutamate ratio. Myocardial alanine levels have been shown to increase in experimental model of diabetes (Scharff and Wool 1966). Furthermore cardiac activity of alanine aminotransferase increases in diabetes (Kazmi and Baquer 1985) and seems to predict coronary heart disease events (Schindhelm et al. 2007). In the non-diabetic CAD patients, alanine was significantly higher in the LV compared to RV (Figure 2B). The data suggests this is unlikely to be due ischemic coronary disease as the alanine/glutamate ratio is similar (Figure 5B). However, although the alanine/glutamate ratio has been used as a marker of anaerobic metabolism during ischemia, its importance in chronic disease has not been fully addressed. Key aspects relating to amino acids ratio(s) is the fact that this ratio will be altered by changes in individual amino acid transporter(s). Therefore we can only speculate that diabetes alters the expression/activity of amino acids transporters and therefore the ratio. The observation that non-diabetic RV also has lower taurine suggests that the basal levels of both amino acids (alanine and taurine) in “relatively” normal RV are lower than corresponding LV. Work to support this suggestion must come from biopsies obtained from normal heart. In diabetic RV of CAD patients, aspartate tends to be higher than non-diabetic RV (Figure 2B). This could partly be due to diabetes stimulating the malate-aspartate shuttle which could improve ATP production (Hadj et al. 2003; Kazmi et al. 1985).

### Female patients with aortic valve stenosis have relatively low metabolic stress compared to male patients

There was no difference in levels of amino acids between left and right ventricles in the male patients (Figure 3). The two chambers of the heart appear to be different in females but not males. The LV of female patients had significantly more glutamate, aspartate and asparagine than corresponding right ventricle, and more glutamate and glutamine than LV of male patients.

The higher concentration of glutamine in the LV of female patients will have effects on transport and metabolism (Rennie et al. 1994). Like taurine, it will influence Na^+^ levels as a result of the increased Na-glutamine symport activity. Glutamine is also metabolically active and acts as a nitrogen donor for the biosynthesis of a number of compounds such as nucleotides and amino acids including glutamate (Rennie et al. 2001). Although glutamate can be used as substrate for energy production especially during metabolic stress, it also has strong antioxidant activity in cardiomyocytes (King et al. 2003; Williams et al. 2001). Due to its cardio-protective effects, glutamate has been used to supplement cardioplegia (Robertson et al. 1984; Rosenkranz et al. 1984, 1986).The total α-amino acids in the LV of female patients was higher than in other ventricles, although this was only significant compared to male LV. This was associated with relatively lower metabolic stress/activity compared to male LV as shown by the alanine/glutamate ratio (Figure 6B).

The higher level of aspartate in LV compared to RV in female patients with AVS (Figure 3B) could have implications for coping with hypertrophy (stress) as this amino acid plays an important role in oxidative phosphorylation and in improving energy production (Hadj et al. 2003).

Overall, this work suggests that LV of female patients with aortic valve stenosis are better prepared to cope with stress compared to the corresponding RV and the LV of male patients. This is consistent with the suggestion that females adapt to pressure overload differently from males and could in part explain the finding that regression of myocardial hypertrophy after aortic valve replacement is faster in females (Petrov et al. 2010).

### Implication for myocardial protection during aortic valve replacement and coronary artery bypass graft surgery

The differences in amino acids between LV and RV could have implications for the extent of ischemia and reperfusion injury during open heart surgery. This of course will depend on the pathology and the amino acid in question. For example higher taurine levels in the LV of CAD non-diabetics and AVS females compared to corresponding RV suggests improved ability to cope with Na^+^ loading and other cellular changes (e.g. oxidative stress). This is more complicated when it comes to α-amino acids. Although it can be argued that higher glutamine, glutamate and aspartate in tissues is also beneficial, higher alanine can be considered as an indicator of increased vulnerability.

The observed differences between the left and right ventricles of patients with either valve stenosis or coronary artery disease and between ventricles of the two pathologies clearly indicate that designing a cardio-protective strategy should take into account these differences. Myocardial protection techniques are largely aimed at preventing (reducing) the cellular changes in metabolites and ions. However, having two different ventricles with two different starting points would require a more thorough consideration of the formulation of cardioplegia. Diabetes in coronary artery disease and gender in aortic valve stenosis are important determinants or remodeling in amino acid content with potential impact on vulnerability to ischemia and reperfusion injury during open heart surgery.
